# Supplementary material for: Color, activity period, and eye structure in four lineages of ants: Pale, nocturnal species have evolved larger eyes and larger facets than their dark, diurnal congeners
Source: PLoS One. 2022 Sep 22;17(9):e0257779. doi: 10.1371/journal.pone.0257779 (PMC9499225; doi:10.1371/journal.pone.0257779)
Supplement: S1 Table — Phylogenies were not available for these genera, so we analyzed the first five species/nominal subspecies from each genus on Antweb (www.antweb.org). Species are listed alphabetically by subfamily, genus, species group, species, and subspecies. High resolution photographs of each species can be viewed at https://www.antweb.org/advSearch.do, then typing in the genus and species name in the advanced search box. Brightness values are given as mean (n) (see text). Relative eye size was calculated as eye area/mesosoma length. (DOCX) [file pone.0257779.s001.docx]

**S1 Table**. Brightness values and relative eye size for dark congeners from the ant genera *Dorymyrmex*, *Iridomyrmex*, and *Temnothorax*. Phylogenies were not available for these genera, so we analyzed the first five species/nominal subspecies from each genus on Antweb (www.antweb.org). Species are listed alphabetically by subfamily, genus, species group, species, and subspecies. High resolution photographs of each species can be viewed at https://www.antweb.org/advSearch.do, then typing in the genus and species name in the advanced search box. Brightness values are given as mean (*n*) (see text). Relative eye size was calculated as eye area/mesosoma length.

| **Species** | **Brightness**  **value** | **Relative**  **eye size** |
| --- | --- | --- |
| **Subfamily Dolichoderinae – genus *Dorymyrmex*** | | |
| *D*. alw-03 | 46.0 (1) | 0.0242 |
| *D. amazonicus* Cuezzo &  Guerrero | 60.7 (1) | 0.0244 |
| *D. antarcticus* Forel | 35.7 (2) | 0.0249 |
| *D. antillanus* Snelling | 43.0 (3) | 0.0234 |
| *D.* az-03 | 46.0 (1) | 0.0242 |
| **Subfamily Dolichoderinae – genus *Iridomyrmex*** | | |
| *I. agilis* Forel | 58.7 (2) | 0.0228 |
| *I. alpinus* Heterick &  Shattuck | 45.5 (2) | 0.0181 |
| *I. anceps* (Roger) | 50.7 (5) | 0.0231 |
| *I. anderseni* Shattuck | 41.3 (1) | 0.0278 |
| *I. angusticeps* Forel | 41.1 (3) | 0.0234 |
| **Subfamily Myrmicinae – genus *Temnothorax*** | | |
| *T. achii* Prebus | 30.0 (1) | 0.0188 |
| *T. acuminatus* Prebus | 40.3 (1) | 0.0187 |
| *T. acutispinosus* Prebus | 43.7 (1) | 0.0224 |
| *T. adjustus* (MacKay) | 34.0 (4) | 0.0184 |
| *T. aeolius* (Forel) | 64.7 (1) | 0.0171 |
