# Supplementary material for: Color, activity period, and eye structure in four lineages of ants: Pale, nocturnal species have evolved larger eyes and larger facets than their dark, diurnal congeners
Source: PLoS One. 2022 Sep 22;17(9):e0257779. doi: 10.1371/journal.pone.0257779 (PMC9499225; doi:10.1371/journal.pone.0257779)
Supplement: Attachment — Submitted filename: Respones to reviewers.docx [file pone.0257779.s004.docx]

Reviewers' comments:

Reviewer's Responses to Questions

**Comments to the Author**

1. If the authors have adequately addressed your comments raised in a previous round of review and you feel that this manuscript is now acceptable for publication, you may indicate that here to bypass the “Comments to the Author” section, enter your conflict of interest statement in the “Confidential to Editor” section, and submit your "Accept" recommendation.

Reviewer #1: All comments have been addressed

Reviewer #2: (No Response)

2. Is the manuscript technically sound, and do the data support the conclusions?

The manuscript must describe a technically sound piece of scientific research with data that supports the conclusions. Experiments must have been conducted rigorously, with appropriate controls, replication, and sample sizes. The conclusions must be drawn appropriately based on the data presented.

Reviewer #1: (No Response)

Reviewer #2: Yes

3. Has the statistical analysis been performed appropriately and rigorously?

Reviewer #1: (No Response)

Reviewer #2: I Don't Know

4. Have the authors made all data underlying the findings in their manuscript fully available?

The [PLOS Data policy](https://urldefense.com/v3/__http:/www.plosone.org/static/policies.action*sharing__;Iw!!IKRxdwAv5BmarQ!ZB6Ve-b97i_Jsm1YVMISX2mPm6CdCX1U_ZF9e2UQDxjrVFBuoi25of54gMajTZSGsC0N8y2g8HvOOBh7RtLotg$) requires authors to make all data underlying the findings described in their manuscript fully available without restriction, with rare exception (please refer to the Data Availability Statement in the manuscript PDF file). The data should be provided as part of the manuscript or its supporting information, or deposited to a public repository. For example, in addition to summary statistics, the data points behind means, medians and variance measures should be available. If there are restrictions on publicly sharing data—e.g. participant privacy or use of data from a third party—those must be specified.

Reviewer #1: (No Response)

Reviewer #2: Yes

5. Is the manuscript presented in an intelligible fashion and written in standard English?

PLOS ONE does not copyedit accepted manuscripts, so the language in submitted articles must be clear, correct, and unambiguous. Any typographical or grammatical errors should be corrected at revision, so please note any specific errors here.

Reviewer #1: (No Response)

Reviewer #2: Yes

6. Review Comments to the Author

Please use the space provided to explain your answers to the questions above. You may also include additional comments for the author, including concerns about dual publication, research ethics, or publication ethics. (Please upload your review as an attachment if it exceeds 20,000 characters)

Reviewer #1: Dear authors,

Thanks for the clarification and changes to the main text. For some reason, I missed the interest in intraspecific variation, which is now much clearer. I had already liked the manuscript in the first round, and I believe this new version is even more precise. Again congratulations on the interesting work.

Reviewer #2: I congratulate the authors by the improved version of the manuscript provided. Most of my first appointments were satisfactorily met, and I just have minor recommendations for this new version, as follows:

Lines 77-79: Given that the authors also didn’t control explicitly for the phylogenetic relationships of the species in their statistical analysis, I would say that they cannot advocate that here they are avoiding completely those issues, as this sentence could suggest. I would suggest a little rephrasing here to avoid the idea that in the present manuscript phylogenetic corrections were explicitly performed.

We have added text to the end of this paragraph to indicate that we did not perform phylogenetic corrections. The added text reads, “In this study, phylogenies were available for all four examined ant genera, such that we could compare eye structure of closely related pale and dark species. However, phylogenetic corrections were not performed”.

Line 93: In “how closely the eye is constructed” I would replace “is constructed” by “develops”.

Changed as suggested

Lines 113-114: This predictive hypothesis need to be reviewed, since the authors did not test the relationship between brightness and activity period for this additional species.

We have revised the last sentence of the paragraph as per the suggestion of the reviewer. The sentence previously read, “Here again, we expected pale body color to be correlated with nocturnal activity as well as eye morphology that enhances visual sensitivity”.

The sentence has been revised to read, “We could not test the correlation between body color and nocturnal activity in these species, but we expect pale body color to be correlated with eye morphology that enhances visual sensitivity”.

Lines 125-128: for the remaining genera considered, the author justify the choice of the species by their phylogenetic relationships, but here for Myrmecocystus the choice was based on body size, and the phylogenetic relationships among the choose pairs of species seems not to follow a clear pattern as in the other genera. What was the criteria to choose those species pairs?

The first paragraph discussing *Myrmecocystus* and species selection has been rewritten for clarity and additional information on species selection. The paragraph now reads, “*Myrmecocystus*: We examined 74 workers from six species (Fig 1). This genus is restricted to North America, and it consists of 29 described species (Snelling 1982, 1976), plus several undescribed and cryptic species (van Elst *et al.* 2021). Species of *Myrmecocystus* display a wide range of body sizes, and all species are size polymorphic. We compared three size-similar species pairs that differed in pigmentation across this range of body sizes: small (*M. christineae* and ***M. yuma***), medium (*M. navajo* and ***M. kennedyi***), and large (*M. mexicanus*-02 and ***M. mendax*-03**). The genus consists of two major clades: most pale species occur in one clade, and the rest of the pale species and all dark species occur in the other clade. The latter clade consists of six smaller clades – one includes the rest of the pale species, and the other five consist of dark species (van Elst *et al.* 2021). Consequently, we could not compare eye structure for phylogenetically adjacent pale and dark species. Instead, we chose both pale and dark species pairs based on a combination of availability of specimens, taxonomic stability, and relative ease of identification”.

Line 329: Don't seems to be the case for many of them (P < 0.05), specially in the case of Box’s M test, but there are also problems with some Levene's tests and Wilk's lambda. I suggest the authors to review this statement and the tests to be sure if the assumptions were really met.

We have reanalyzed the data for *Temnothorax* to account for the statistics not meeting the model assumptions for the homogeneity of regression slopes. Data were transformed again to help meet the assumptions, but problems still existed. Consequently, we redid the analysis in a slightly different way such that the assumptions were met. Additionally, note that we use an alpha value of *P* < 0.001 to denote significance for the Box’s M test, which is in accord with discussions of this statistic – references are added in the text to support this assertion. We also added a discussion regarding *Veromessor* and model assumptions. Text regarding the analysis for *Veromessor* is presented following the text for *Temnothorax*.

We have changed the text under the subheading “Eye area, facet number, and facet diameter” to describe our statistical methods for *Temnothorax*. The sentence, “Dependent variables met both assumptions for all four genera (Table 3)”, has been replaced by the following, “Box’s M test for homogeneity of variance-covariance matrices is not robust to violations of this assumption such that an alpha level of *P* < 0.001 is recommended (Hahs-Vaughn 2017; Tabachnick & Fidell 2001). *Myrmecocystus* and *Aphaenogaster* met both assumptions. *Temnothorax* met the homogeneity of variance-covariance assumption but not the assumption for homogeneity of regression slopes because the slopes for eye area differed across species (*P* = 0.007) (Table 3). Consequently, we reran the model after deleting eye area, and the homogeneity of regression slopes assumption was met using the two other dependent variables (facet number, facet diameter) (Table 3). We then analyzed variation in eye area across species separately using a one-way ANOVA. The dependent variable in this model was relative eye size, calculated as eye area/mesosoma length, which standardized eye area for body size (see above). *Veromessor* did not meet the homogeneity of variance-covariance assumption, so we used Pillai’s trace instead of Wilks’lambda to evaluate multivariate significance (Olson 1797). The Levene’s test for *Veromessor* was also significant for the regression slopes comparison and in the final model. Consequently, we decreased the *P* value for post-hoc tests to *P* < 0.01 (Tabachnick & Fidell 2001).

The following references regarding these statistical methods were added to the text.

Hahs-Vaughn DL. 2017. *Applied multivariate statistical concepts*. Routledge, New York.

Olson CL. 1797. Practical considerations in choosing a MANOVA test statistic: a rejoinder to Stevens. *Psychological Bulletin* **86**, 1350–1352.

Tabachnick BG, Fidell LS. Using multivariate statistics. 6th ed. Upper Saddle River, New

Jersey: Pearson; 2014.

Accordingly, we have also added information regarding *Temnothorax* to the caption of Table 3 and to Table 3 itself. To the end of the caption we have added, “For *Temnothorax*, the first line is for all three variables, the second line is the model after deleting eye area (see text); values in the second line for the Levene’s test are for facet number and facet diameter, respectively”. We also note in this Table legend that Pillai’s trace was used instead of Wilks’ lambda for the multivariate test for *Veromessor,* which was also noted in the above revised methods.

The results are basically the same as before, but the section on *Temnothorax* has been revised to reflect the different analysis that is now used. The section for *Temnothorax* now reads, “Eye structure (facet number, facet diameter) differed among species of *Temnothorax* after controlling for mesosoma length (MANCOVA: Wilks’ λ= 0.054, F_4,48_ = 39.5, *P* < 0.001). The tests of between-subject effects demonstrated that both facet number and facet diameter differed across species (facet number: F_2,25_ = 20.8, *P* < 0.001; mean *D*: F_2,25_ = 53.4, *P* < 0.001; Fig 8). Based on estimated marginal means, pairwise comparisons across all species pairs using a LSD test showed that mean *D* was larger for the pale *T.* sp. BCA-5 than for the two dark congeners, and that facet number was higher in ***T*. *tricarinatus*** than for both ***T. neomexicanus*** and *T*. sp. BCA-5 (Fig 8). Relative eye area also differed across species (one-way ANOVA: F_2,26_ = 44.0, *P* < 0.001), with size being largest for *T.* sp. BCA-05, intermediate for ***T. tricarninatus***, and smallest for ***T. neomexicanus*** (LSD test, *P* < 0.05; Fig 8). Mean *D* (using estimated marginal means) was 1.23× larger for *T.* sp. BCA-5 (17.76 μm) compared to ***T. neomexicanus*** (14.43 μm) and 1.32× larger than that for ***T. tricarinatus*** (13.42 μm) (Table 1).

Mesosoma length was an important covariate in the model (Wilks’ λ = 0.546, F_2,24_ = 9.97, *P* < 0.001), and tests of between-subjects effects differed for facet number (F_1,25_ = 20.8, *P* < 0.001), but not for mean *D* (F_1,25_ = 3.9, *P* = 0.058). Eye area and facet number increased with body size within all three species, while mean *D* increased with body size for *T*. sp. BCA-5 and ***T. tricarinatus***, but it decreased with body size for ***T. neomexicanus*** (Fig 8).

In regard to *Veromessor*, the results and graphs have been rewritten accordingly using an alpha value of *P* < 0.01

Lines 366-368: Here again it is not clear what is the meaning of the significant ANCOVA. The manuscript is large and refers to many different results and tests, so if the reader should return to the methods section every time a statistical test is present in the results section to check what exactly the test means, the reading would be exhausting. I suggest the authors to present the results in a more straightforward way, indicating exactly what the result of the test means (like “the test suggest that individuals differ in size (results of the test)” or “the test indicates that the diameter of the ocellus is greater in pale than dark species (results of the test)”. Although the authors already improved those issues that I pointed in the first review, which I comply for the effort, it could still be improved in some instances as this one.

The first sentence of this paragraph has been rewritten for additional clarity and now reads, “Diameter of the anterior ocellus differed across species after controlling for mesosoma length (ANCOVA: tests of between-subject effects; F_7,98_ = 69.6, *P* < 0.001; Fig. 6), but the species × mesosoma length interaction term was not significant (F_7,91_ = 0.96, *P* = 0.47)”.

Lines 446-451: The same as the previous comment. I would suggest to rewrite this sentence as “Genus has an important effect on Δϕ (F3,32 = 10.1, P < 0.001). Although the isolated effect of activity period over Δϕ showed a weak effect (F1,32 = 4.0, P = 0.055), the interaction of genus × activity period showed a strong effect (F3,32 = 7.3, P < 0.001), which indicates that the four genera differed in the direction and magnitude of differences in Δϕ”.

This sentence has been rewritten as suggested by the reviewer. The paragraph now reads, “Genus has an important effect on Δ*ϕ* (ANCOVA: F_3,32_ = 10.1, *P* < 0.001). Although the isolated effect of activity period over Δ*ϕ* showed a weak effect (F_1,32_ = 4.0, *P* = 0.055), the interaction of genus × activity period showed a strong effect (F_3,32_ = 7.3, *P* < 0.001), which indicates that the four genera differed in the direction and magnitude of differences in Δ*ϕ*. Across all species, values of Δ*ϕ* ranged from 3.5–7° (Fig 10).

Lines 456-459: Another rewriting suggestion to inform the results in a more straightforward manner and reduce text length: “Mesosoma length was an important covariate in the above model (F1,31 = 5.4, P = 0.026), a fact largely influenced by Δϕ decreasing in larger workers of Temnothorax and Veromessor, and not varying with mesosoma length within species of Myrmecocystus and Aphaenogaster (Fig 10).”.

To reinforce, those suggestions to rewrite statements describing the results should be taken only as suggestions, the authors can decide to not follow them or to apply them throughout the results section if they agree that it would improve the readability of the manuscript.

I also believe that the comment/edit by the reviewer makes the manuscript clearer. We have made the change as suggested to this comment. We have also edited other paragraphs that discuss mesosoma length as a covariate so as to have similar construction.

Lines 466-468: There is no strong effect (difference in p based on P-value) between pale and dark species in those genera except in Myrmecocystus, I suggest the authors to take a look on it.

We have edited the second part of this paragraph as suggested. It now reads: “Pale species had the larger mean *ρ* in *Myrmecocystus* (t-test: t_8 df_ = -8.9, *P* < 0.001), while values did not differ between pale and dark species in the other three genera (*Veromessor*: t_8_ = -0.3, *P* = 0.74; *Temnothorax*: t_4_ = -1.7, *P* = 0.16; *Aphaenogaster*: t_8_ = -2.2, *P* = 0.058). Across genera *ρ* was highest for *Aphaenogaster*, intermediate for *Temnothorax* and *Veromessor*, and lowest for *Myrmecocystus* (Tukey’s HSD test, *P* < 0.05; Fig 11)”.

Lines 545-546: The relationship between body color and activity period does not support the hypothesis that activity pattern produces strong selection patterns in eye structure, please rewrite this statement.

We are not sure where the reviewer is going with this? We believe that activity period is the precursor to color and eye structure, such that activity patterns is the first trait to drive these other changes. We have rewritten this sentence slightly so that it now reads, “The above two patterns support the hypothesis that activity pattern can produce strong selection pressure on body coloration and eye structure”.

Line 599: V. smithi should be in bold face, right?

Yes, it should be bold font, and it has been changed.

Line 614: The symbol for micrometers is not correct, please take a look on it.

This has been changed to μm

Lines 621-623: It is not clear here which genera the authors are referring by “latter three genera”.

This sentence has been changed for better clarity. It previously read, “The lack of consistent patterns across the latter three genera likely …”. It now reads, “The lack of consistent patterns across species of *Aphaenogaster*, *Temnothorax* and *Veromessor* likely …”

Line 676: There are images of M. pyramicus available in AntWeb.
Yes, we are aware that there images of *M. pyramicus* on ANTWEB. For all other species that we included, we had two or more long series of workers to examine for ocelli and variation in presence/absence across all sizes, etc. ANTWEB only has photos of individual workers of *M. pyramicus* and we thus preferred to omit this species because of the limited sample size and number of colonies to examine.

Tables

Table 1: I suggest the authors to indicate in the table that “facet diameter” is the variable D, since this is how they refer to this variable throughout most of the text.

*D* has been added to subheading in Table 1

Table 4: Please replace Mauchley’s by Mauchly’s.

Mauchley’s changed to Mauchly’s

7. PLOS authors have the option to publish the peer review history of their article ([what does this mean?](https://urldefense.com/v3/__https:/journals.plos.org/plosone/s/editorial-and-peer-review-process*loc-peer-review-history__;Iw!!IKRxdwAv5BmarQ!ZB6Ve-b97i_Jsm1YVMISX2mPm6CdCX1U_ZF9e2UQDxjrVFBuoi25of54gMajTZSGsC0N8y2g8HvOOBhAm2_pwQ$)). If published, this will include your full peer review and any attached files.


If you choose “no”, your identity will remain anonymous but your review may still be made public.


**Do you want your identity to be public for this peer review?** For information about this choice, including consent withdrawal, please see our [Privacy Policy](https://urldefense.com/v3/__https:/www.plos.org/privacy-policy__;!!IKRxdwAv5BmarQ!ZB6Ve-b97i_Jsm1YVMISX2mPm6CdCX1U_ZF9e2UQDxjrVFBuoi25of54gMajTZSGsC0N8y2g8HvOOBhOfLEqJw$).

Reviewer #1: No

Reviewer #2: No

[NOTE: If reviewer comments were submitted as an attachment file, they will be attached to this email and accessible via the submission site. Please log into your account, locate the manuscript record, and check for the action link "View Attachments". If this link does not appear, there are no attachment files.]

While revising your submission, please upload your figure files to the Preflight Analysis and Conversion Engine (PACE) digital diagnostic tool, [https://pacev2.apexcovantage.com/](https://urldefense.com/v3/__https:/pacev2.apexcovantage.com/__;!!IKRxdwAv5BmarQ!ZB6Ve-b97i_Jsm1YVMISX2mPm6CdCX1U_ZF9e2UQDxjrVFBuoi25of54gMajTZSGsC0N8y2g8HvOOBjvALxN5Q$). PACE helps ensure that figures meet PLOS requirements. To use PACE, you must first register as a user. Registration is free. Then, login and navigate to the UPLOAD tab, where you will find detailed instructions on how to use the tool. If you encounter any issues or have any questions when using PACE, please email PLOS at [figures@plos.org](mailto:figures@plos.org). Please note that Supporting Information files do not need this step.
